# The effects of sequencing platforms on phylogenetic resolution in 16 S rRNA gene profiling of human feces

**DOI:** 10.1038/sdata.2018.68

**Published:** 2018-04-24

**Authors:** Tae Woong Whon, Won-Hyong Chung, Mi Young Lim, Eun-Ji Song, Pil Soo Kim, Dong-Wook Hyun, Na-Ri Shin, Jin-Woo Bae, Young-Do Nam

**Affiliations:** 1 Department of Life and Nanopharmaceutical Sciences and Department of Biology, Kyung Hee University, Seoul 130-701, Republic of Korea; 2 Research Group of Gut Microbiome, Division of Nutrition and Metabolism Research, Korea Food Research Institute, Sungnam 463-746, Republic of Korea; 3 Korea University of Science and Technology, Daejeon 305-350, Republic of Korea

**Keywords:** Classification and taxonomy, Metagenomics, Sequencing

## Abstract

High-quality and high-throughput sequencing technologies are required for therapeutic and diagnostic analyses of human gut microbiota. Here, we evaluated the advantages and disadvantages of the various commercial sequencing platforms for studying human gut microbiota. We generated fecal bacterial sequences from 170 Korean subjects using the GS FLX+ (V1–4), Illumina MiSeq (V1–3, V3–4 and V4), and PacBio (V1–9) systems. Comparative analyses revealed that the PacBio data showed the weakest relationship with the reference whole-metagenome shotgun datasets. The PacBio system generated sequences with a significantly higher level of deletions than datasets generated by other platforms, with an abnormally high proportion of sequences assigned to the phylum *Proteobacteria*. Low sequencing accuracy and low coverage of terminal regions in public 16 S rRNA databases deteriorate the advantages of long read length, resulting in low taxonomic resolution in amplicon sequencing of human gut microbiota.

## Background & Summary

In microbial ecology, next-generation sequencing (NGS) followed by computational analysis has become routine practice for phylogenetic analysis of bacterial communities in various ecosystems. Accordingly, in a clinical context, our understanding of the human microbiome is expanding, with regard to both the spatiotemporal variation of microbiota in human body sites^1^ and the complex interactions between the microbiome and host factors (e.g., diet, genomic or metabolic phenotypes, and immune responses)^2–4^. The advantages of cost-effective and large-scale multiplexing analyses have focused attention on their potential diagnostic applications for dysbiosis and risk of disease^5^ and the development of personalized medicine^6,7^.

Since Sogin *et al.* first used massively parallel tag sequencing to explore deep-sea microbial diversity^8^, Roche’s 454 platform has been the dominant sequencing platform for amplicon sequencing spanning microbial 16 S rRNA gene fragments^9^. The cost-effective 454 FLX pyrosequencer, used in combination with several bioinformatics tools^10–12^ and denoising algorithms^13,14^, offers a robust approach to microbial community profiling. More recently, however, the 454 platform has been overshadowed by the emergence of other commercially available NGS instruments (Supplementary Fig. 1). For instance, the Illumina MiSeq platform has increasingly outpaced the 454 systems, mainly due to its much higher throughput (up to 25 million reads per run) and continuously improving read length (50, 150, 250, and 300 bp for single-read or paired-end reads) (http://www.illumina.com). Although the 454 platform provides reliable long sequence reads that are still favored in particular research and clinical fields, it will not remain the platform of choice for amplicon sequencing because Roche shut down its NGS research programs in 2016.

The high base calling accuracy and quantitative power of Illumina platforms allow deeper estimation of the environmental 16 S rRNA gene profiles^15,16^; however, the relatively short read length of Illumina-generated sequences can reduce the resolution of taxonomic annotation against reference databases^17,18^. Several recent studies demonstrate that sequence read length, along with the specific combination of primer pairs used for short-read amplicon sequencing, can substantially affect the accuracy and sensitivity of taxonomic discrimination, as well as estimates of taxon abundance^19,20^. Therefore, high precision and adequate read length covering entire 16 S rRNA region are required for the next generation of high-throughput sequencing technologies. As an alternative, the Pacific Biosciences (PacBio) Single Molecule, Real-Time (SMRT) DNA sequencing system has become available for microbial phylogenetic profiling. This method uses circular consensus sequencing (CCS), in which DNA polymerase repeatedly replicates hairpin-ligated amplicons, resulting in longer sequence reads (average >10 kb; http://www.pacb.com) and reduced error rates^21^. Few studies have analyzed the performance of the PacBio platform in the context of full-length 16 S rRNA gene sequencing^20,22,23^. However, neither the use of synthetic microbial communities that normally comprise 20 to 30 microbial species nor the limited sample size makes the PacBio system a solid choice for practical 16 S rRNA gene sequencing of environmental samples.

Here, we sought to identify the quantitative and qualitative features of human gut microbiota according to the practical use of primer combinations for each sequencing platform. Using whole-metagenome shotgun sequencing of human fecal microbiome as an unbiased standard for microbial community structure, we generated human fecal bacterial sequences from 170 Korean subjects using the 16 S rRNA regions most frequently targeted by commercial NGS platforms [GS FLX+ (V1-4), Illumina MiSeq (V1–3, V3–4 and V4) and PacBio CCS (V1–9)] and compared the advantages and disadvantages of each platform for studies of human gut microbiota (Data Citation 1, Data Citation 2, Data Citation 3, Data Citation 4, Data Citation 5 and Data Citation 6, Table 1). Overall, the PacBio dataset showed the most distant relationship with the shotgun data, which represents the standard microbial community in human feces. In addition, the abundance patterns of bacterial taxa in the PacBio dataset were skewed toward the phylum *Proteobacteria* (particularly the family *Enterobacteriaceae*). These results are not the direct consequence of low throughput and/or differences in read length between the query and subject sequences, as evidenced by the close similarity of taxonomic profiles among the sub-sampled datasets and between the full-length and *in silico*-generated partial sequences of the PacBio datasets, respectively. Instead, the results reveal that the indel errors (especially deletion) are the main driver of differences in the gut microbial community profiles obtained from the PacBio dataset.

## Methods

### Study participants and specimen collection

A total of 172 healthy participants (60 males and 112 females; mean age: 73±8.7 years) were enrolled for fecal sample collection in South Korea. Fecal samples from participants were collected at local community health centers, transported to the laboratory, and stored at −80 °C until analysis. The study protocol was approved by the Institutional Review Boards of Korea Centers for Disease Control and Prevention (IRB file No. 2015-02-EXP-05-3C-A). Written informed consent was obtained from all participants.

### DNA extraction from the fecal samples and 16 S rRNA gene sequencing

Fecal bacterial DNA was extracted using the QIAamp Fast DNA Stool Mini Kit (Qiagen, Inc., Valencia, CA, USA) and the extracted DNA further purified using the G-spin genomic extraction kit (iNtRON Biotechnology, Republic of Korea). The elution buffer volumes used in the final step of the two protocols were 100 μL and 50 μL, respectively. The 16 S V1–3 libraries were prepared using the NEXTflex 16 S V1–3 Amplicon-Seq kit (Bioo Scientific, Austin, TX, USA). The primers included in the NEXTflex™ 16 S V1–3 PCR I Primer Mix (Forward, 5′- CTC TTT CCC TAC ACG ACG CTC TTC CGA TCT AGA GTT TGA TCC TGG CTC AG-3′, Reverse 5′- CTG GAG TTC AGA CGT GTG CTC TTC CGA TCT GTA TTA CCG CGG CTG CTG G-3′) were used for 16 S V1–3 amplification. The 16 S V4 libraries were prepared using the NEXTflex 16 S V4 Amplicon-Seq Kit (Bioo Scientific). The NEXTflex™ 16 S V4 Forward Primer (5′- AAT GAT ACG GCG ACC ACC GAG ATC TAC ACT ATG GTA ATT GTG TGC CAG CMG CCG CGG TAA-3′) and NEXTflex™ 16 S V4 Reverse Primer Barcode (5′- CAA GCA GAA GAC GGC ATA CGA GAT XXX XXX XXX XXX AGT CAG TCA GCC GGA CTA CHV GGG TWT CTA AT-3′) were used for 16 S V4 amplification. The 16 S V3–4 regions were amplified using the 16 S Amplicon PCR Forward Primer (5′- TCG TCG GCA GCG TCA GAT GTG TAT AAG AGA CAG CCT ACG GGN GGC WGC AG-3′) and 16 S Amplicon PCR Reverse Primer (5′- GTC TCG TGG GCT CGG AGA TGT GTA TAA GAG ACA GGA CTA CHV GGG TAT CTA ATC C-3′)^24^. Library preparation of the 16 S V3–4 PCR products was performed using Nextera XT Index (Illumina, San Diego, CA, USA). The 16 S V1-4 libraries were prepared according to the GS FLX+ Library Prep guide. The 16 S universal primers 27 F (5′- GAG TTT GAT CMT GGC TCA G-3′) and 800 R (5′- TAC CAG GGT ATC TAA TCC-3′) were used to amplify 16 S V1-4 regions. For PacBio sequencing, primers 27 F (5′- AGR GTT YGA TYM TGG CTC AG-3′) and 1492 R (5′- RGY TAC CTT GTT ACG ACT T-3′) were used to amplify full-length 16 S rRNA genes. The full-length PacBio 16 S libraries were constructed using the 10- kb SMRTbell Template Prep Kit 2.0.

The 16 S V1–3, V3–4, and V4 libraries for each sample were sequenced on the MiSeq platform using the paired-end 2×300 bp reagent kit, according to the manufacturer's instructions (Illumina, San Diego, CA, USA). The sequencing for the 16 S V1-4 region was performed using a Genome Sequencer FLX+ system (Roche Diagnostics, Basel, Switzerland). The 16 S full-length PacBio libraries were sequenced on a PacBio RS II platform (Pacific Biosciences, Menlo Park, CA, USA) using SMRT P6-C4 chemistry.

### Metagenomic shotgun sequencing

We selected 19 individuals among the participants for metagenomic shotgun sequencing. The sequencing library was prepared by random fragmentation of the DNA samples using Accel-NGS 2 S PCR-Free Library Kit (350 bp insert) by following the manufacturer's instruction (Swift Biosciences, Ann Arbor, Michigan, USA). All the prepared libraries were qualified using qPCR according to the Illumina qPCR Quantification Protocol Guide. Illumina TruSeq SR Cluster Kit v4 reagents was used to generate cluster from the resulting libraries and rapid runs were conducted on the HiSeq 2500 platform using TruSeq SBS Kit v4 reagents (Illumina, San Diego, California, USA). As results, we obtained at least 35 million of paired-end reads (2×250 bp) for each sample.

### NGS data processing

#### GS FLX+ sequencing

Before quality filtration, reads from each sample were split according to a given barcode sequence. To exclude poor-quality sequences and/or sequencing errors, sequences containing more than one ambiguous base call, those with errors in the barcode or primer regions, those with average quality scores<25, and those shorter than 200 bp in length, were removed. All of these processing steps were performed using QIIME 1.9.0 (ref. 12). To minimize a type of platform-dependent error, homopolymorphic error, denoising was conducted using Acacia (ver. 1.53)^25^ and default parameters.

#### MiSeq sequencing

Low-quality reads were filtered using Trimmomatic (ver. 0.35)^26^, with a minimum read length of 150 bp and Nextera adapter sequence trimming options. Reads that passed the filtering process for both paired reads were used for downstream analysis. The filtered paired-end reads were joined into single-sequence reads using the paired-end read merger PEAR (ver. 0.9.8)^27^ and default parameters. To exclude anomalously joined reads, reads that were too short or too long were excluded according to the expected size of each targeting region: 463–553 bp for region V1–3, 438–469 bp for region V3–4, and 286–298 bp for region V4. The ranges were determined according to the sequence length distribution of the targeted regions, which were extracted from the Greengenes (GG) database. The minimum and maximum lengths, excluding 1% of outliers, were accepted as the expected sizes of targeted regions.

#### PacBio sequencing

CCS error correction was performed with a minimum 8-fold of reads of insert for raw PacBio sub-reads using the SMRT analysis pipeline (ver. 2.3)^28^. To select full-length 16 S rRNA sequences, CCS reads were filtered according to the following criteria: i) inclusion of the forward primer and ii) 1,300 bp minimum length. If a reverse primer was detected in the middle of a read, it was trimmed at that position.

#### 16 S rRNA gene extraction from shotgun sequencing

To extract 16 S rRNA gene fragments from metagenomic sequences, reads were mapped to the GG database (included in QIIME 1.9.0) using BWA (ver. 0.7.15)^29^. From the BAM-formatted aligned file, the aligned reads were selected using an in-house script that was designed to screen out unmapped reads. The reads were then joined using PEAR, with default parameters. Reads shorter than 200 bp were excluded from downstream analysis.

### Sequence analysis

After read quality filtering, the remaining sequences were processed using QIIME 1.9.0. Chimeric sequences were excluded from the quality-filtered sequences using USEARCH software. Operational taxonomic units (OTUs) were clustered using the open reference OTU picking method (at 97% sequence similarity) using UCLUST software. Singleton OTUs were not included in further analyses. A representative sequence for each OTU was selected, and aligned with the GG-provided reference sequences using PyNAST or the SILVA 123 QIIME compatible database. A phylogenetic tree of the aligned sequences was then constructed using FastTree.

### Simulation study for platform comparison

As the source of data for the simulation study, we chose a set of full-length 16 S rRNA gene sequences from a non-redundant set in the GG database (gg_13_8_99.fasta). The primer sequences used for DNA extraction were BLASTed against to the database sequences. The 46,617 sequences were selected by checking the existence of all primers targeting the following regions: V1–9 for PacBio; V1–4 for GS FLX+; and V1–3, V3–4, and V4 for Illumina. This process yielded mock sequences covering the five targeted regions.

To compare the sequences between targeted regions, we computed the best-hit per query and obtained a pairwise alignment using BLASR^30^ (options: -bestn 1 -m 5 -minPctIdentity 80 -maxScore 500). From the pairwise alignment, the following three values were computed: sequence identity considering all types of differences (mismatch, insertion, and deletion), sequence identity counting mismatch only, and the insertion/deletion ratio. The correlation between pairwise similarities of two targeted regions was computed and plotted using the function ggpairs in the R library ggplot2 (ref. 31).

The effect of the targeted region on OTU clustering was measured by precision (or positive prediction value) and recall (or sensitivity). Bootstrap sets (*n*=100) of mock sequences were built for each targeted region. To make a set of mock sequences, 10,000 sequences were randomly chosen from the targeted region. Each set of mock sequences was clustered using the same OTU picking method, as described in the subsection of sequence analysis. Sequences missing from a cluster (false-negatives), as well as added sequences (false-positives), were counted by comparison with the cluster originally defined by the GG database. The precision of this experiment was defined as the proportion of truly associated sequences in the computed cluster. Recall was defined as the proportion of sequences that were successfully found in the GG-defined cluster.

### Abundance of insertion and deletion per sequencing platform

Insertion and deletion ratios of the five sequencing libraries representing three sequencing platforms were computed by comparison with the GG database. As for the simulation study described above, the best-hit sequence was searched against non-redundant sequences (gg_13_8_99.fasta), to yield a pairwise alignment for each sequence read. The numbers of inserted and deleted bases were normalized against read length.

### Statistical analysis

Statistical analyses were performed using GraphPad Prism version 5.0 for Windows (GraphPad Software, La Jolla, CA, USA). Comparisons between multiple samples were conducted by analysis of variance (ANOVA), followed by Tukey’s *post-hoc* test (*P<0.05, **P<0.005, and ***P<0.001). The lines, boxes, and whiskers in the box-plot diagrams represent the median, first and third quartiles, and min-to-max distribution of replicate values, respectively. Values and scattered dots in the bar graphs represent the means±SEM and individual replicates, respectively.

## Data Records

We applied the practical use of primer combinations for each sequencing platform to characterize the quantitative and qualitative features of human gut microbiota. The datasets supporting the conclusions of this article are available (Data Citation 1, Data Citation 2, Data Citation 3, Data Citation 4, Data Citation 5 and Data Citation 6). Data from publically available PacBio sequences (vulture gut metagenome) are available (Data Citation 7).

## Technical Validation

### Overall comparison of NGS datasets

**Figure 1:**
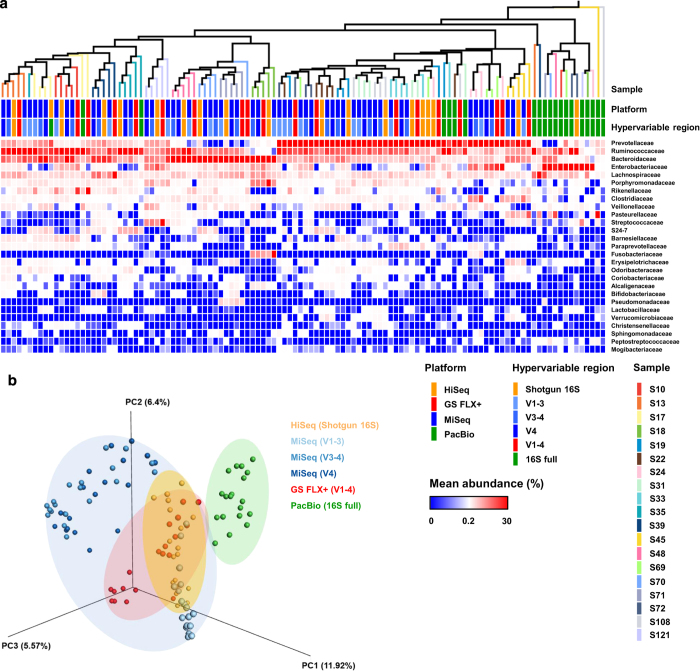
Comparative analysis of human fecal bacterial NGS datasets. Fecal samples collected from 19 human subjects were sequenced using the indicated platforms: GS FLX+ (V1–4, red), Illumina MiSeq (V1–3, light blue; V3–4, blue; V4, dark blue), and PacBio CCS (V1–9, green). Whole-genome shotgun sequences generated by Illumina HiSeq (Shotgun 16 S, orange) were included as a reference for community structure without amplification bias. (**a**) The sequence data were clustered using a UPGMA dendrogram based on the Bray-Curtis dissimilarity matrix, and samples from the same individual are shown in the same color. The relative abundances of bacterial taxa are displayed as a heatmap over 27 families (>1% relative abundance). (**b**) The sequence data were clustered by principal component analysis.

Previous studies of mock community-based sequencing reveal that the high error rate of PacBio-generated sequences can be reduced to values as low as those generated by the 454 or Illumina systems by increasing the CCS coverage^22,23^. Accordingly, we assessed the suitability of PacBio system for amplicon sequencing of human gut microbiota. Using practical combinations of primer sets for each sequencing platform, we generated GS FLX+ (V1–4), Illumina MiSeq (V1–3, V3–4 and V4), and PacBio CCS (V1–9) datasets from fecal samples from 19 Korean subjects. In addition, we included whole-metagenome shotgun sequences (Illumina HiSeq) from the 19 fecal samples as references for community structure, with no amplification bias^19,22^. For unbiased estimation of taxonomic profiles from the shotgun data, we applied cellular relative abundance by generating an *in silico* 16 S rRNA gene dataset^32^. The UPGMA dendrogram based on the Bray-Curtis dissimilarity matrix showed that most samples, including the shotgun data, clustered according to sample type (i.e., samples from the same individual) regardless of the platform or combination of primer pairs (Fig. 1a). However, the PacBio samples showed marked separation from those generated by the other platforms. Principal coordinates analysis confirmed the distant clustering of the PacBio samples (Fig. 1b). Moreover, correlation analysis of bacterial abundance between the sequence datasets (27 families, >1% relative abundance) revealed that the PacBio datasets showed the weakest relationship to the shotgun data (Table 2).

### Sequencing performance of GS FLX+, MiSeq, and PacBio in human fecal samples

We next examined the advantages and disadvantages of each sequencing platform for studying human gut microbiota. To this end, we expanded the cohort size for GS FLX+ (V1-4) and Illumina MiSeq (V4) datasets to include 169 Korean subjects. We then divided the MiSeq V4 dataset into three enterotypes based on Euclidean distance^33^ and subjected high ranking samples in each enterotype (*n*=29) to full-length 16rRNA gene sequencing on the PacBio platform. This study design was based on our interest in preferentially targeting 16 S rRNA regions for PCR-amplification in each platform and the comparable cost of generating each type of dataset. In contrast to the MiSeq V4 dataset, which contained a larger number of sequence reads per sample (mean, 78,466 reads/sample) with the shortest average read length (291 bp), the PacBio dataset contained the smallest number of sequence reads per sample (mean, 2,649 reads/sample) with the longest average read length (1,481±27 bp) (Table 3). The features of each platform-generated dataset are reflected by rarefaction and alpha diversity analyses of human gut microbiota, and the MiSeq V4 dataset had a relatively highly saturated rarefaction curve despite having the highest values in terms of richness indices (Supplementary Figs 2 and 3). The PacBio dataset had a rarefaction curve with a steep slope, and significantly lower values for diversity, richness, and evenness indices than those in other datasets.

### Platform comparison for taxonomy profiling

**Figure 2:**
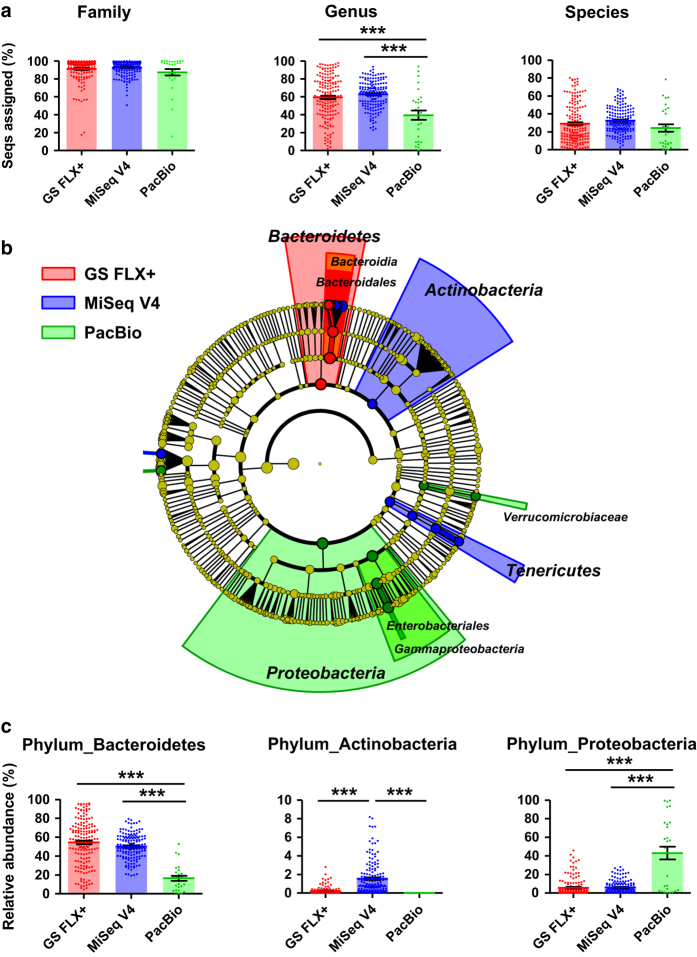
Taxonomy profiles in each platform-generated datasets. (**a**) The proportion of assigned sequences from the GS FLX+ (*n*=169, red), MiSeq V4 (*n*=169, blue), and PacBio (*n*=29, green) datasets was determined by assigning the sequences to the GG 16 S rRNA gene sequence database, and are represented at the family, genus, and species levels. (**b**) The abundance patterns of bacterial taxa in each dataset were analyzed using the linear discriminant analysis effect size (LEfSe) circular cladogram. The discriminant taxa for the GS FLX+, MiSeq V4, and PacBio datasets are shown in red, blue, and green, respectively. (**c**) Relative abundances of the phylum *Bacteroidetes*, *Actinobacteria* and *Proteobacteria* in OTU tables for each dataset are represented as bar graphs. Data were analyzed by ANOVA followed by Tukey’s post hoc test (*P<0.05, **P<0.005, and ***P<0.001).

To determine whether the difference in read lengths affects the ability to assign a sequence to the lower ranks of taxonomic lineages, we analyzed the proportion of assigned sequences from the GS FLX+ (*n*=169), MiSeq V4 (*n*=169), and PacBio (*n*=29) datasets at the family, genus, and species levels. The GG 16 S rRNA gene sequence database was used as reference taxonomy because of its robust performance in analysis of human gut sequences and the specific taxon lineages that allow species-level description^23,34^. We observed difference in taxonomic resolution among datasets at the family or species level (Fig. 2a). However, at the genus level, the PacBio dataset had the lowest proportion of assigned sequences (mean 59.3, 62.7, and 39.4% for GS FLX+, MiSeq V4 and PacBio datasets, respectively), suggesting that the greater average length of the PacBio-generated reads was not associated with higher taxonomic resolution in human fecal bacterial sequences.

The pattern of abundance of bacterial taxa within each dataset was identified using the linear discriminant analysis effect size (LEfSe) method^35^. The LEfSe circular cladogram indicated that phyla *Bacteroidetes*, *Actinobacteria*, and *Proteobacteria* were the discriminant taxa for the GS FLX+, MiSeq V4, and PacBio datasets, respectively (Fig. 2b). We subsequently compared the relative abundance of these taxa in OTU tables, and found that phylum *Bacteroidetes*, the predominant taxon in the GS FLX+ and MiSeq V4 datasets, constituted a significantly smaller proportion of the PacBio dataset (Fig. 2c). In addition, the PacBio dataset contained an abnormally high proportion of sequences assigned to the phylum *Proteobacteria*. In the subject-standardized datasets, we observed high reproducibility in the alpha diversity analysis, proportion of assigned sequences, and abundance patterns of bacterial taxa (Table 4, and Supplementary Fig. 4 and 5). Collectively, these data suggest that the current PacBio CCS is still less robust than other sequencing platforms in terms of characterizing the complex microbial 16 S rRNA gene profiles in human gut microbiota.

### Error profiles of PacBio-generated human fecal 16 S rRNA sequences

**Figure 3:**
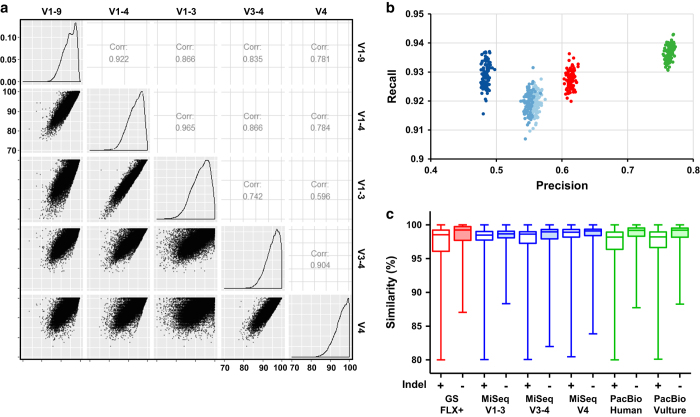
Relationship between sequence read length and assignment to database. (**a** and **b**) Sequences (*n*=10,000) covering the V1–4 (red), V1–3 (light blue), V3–4 (blue), V4 (dark blue), and V1–9 (green) regions of the 16 S rRNA gene were extracted *in silico* from the GG 16 S rRNA gene database. (**a**) Pairwise similarities between targeted regions are represented as similarity distributions (lower left), similarity histograms (diagonal), and correlation coefficient values (upper right). (**b**) Accuracy of phylogenetic reconstruction per targeted region. Precision (x-axis) and recall (y-axis) were calculated based on 100 randomly chosen bootstrap replications. (**c**) Distribution of sequence (30,000 sub-sampled) similarities, including and excluding indel errors, between data generated by GS FLX+, Illumina MiSeq, and PacBio, and data from publically available PacBio sequences.

Pairwise similarity between query and subject 16 S rRNA sequences is expected to vary according to the length and/or region of the query sequences. To assess the pairwise similarity of different 16 S rRNA regions, we generated *in silico* V1–9, V1–4, V1–3, V3–4, and V4 datasets randomly extracted from the GG 16 S rRNA gene database and evaluated the statistical relationships of pairwise similarities between the GG 16 S rRNA-extracted datasets. The correlation coefficient analyses clearly showed a positive correlation between sequence read length and pairwise similarity (Fig. 3a). We next assessed whether the read length and/or the region of 16 S rRNA affected phylogenetic reconstruction. To this end, we evaluated both precision (i.e., prediction value for non-false positive phylogenetic position) and recall (i.e., bootstrap sensitivity) from the GG 16 S rRNA-extracted datasets. The precision ratio was highest in the V1–9 dataset, representing a striking positive relationship between read lengths of extracted datasets and the precision of phylogenetic construction (Fig. 3b). These simulation results, together with the low taxonomic resolution of the PacBio-generated sequences, suggest that longer reads are advantageous for taxonomic discrimination, but that the PacBio generated sequences, which consist of abundant full-length 16 S rRNA sequences, may contain sequencing errors.

Because GG 16 S rRNA gene database contains fragmentary 16 S rRNA gene sequences, it has low coverage of the V1 and/or V9 regions (Supplementary Fig. 6). To determine whether the low taxonomic resolution of the PacBio data resulted from differences in read length between the query and reference sequences (e.g., assignment of the PacBio-generated full-length 16 S rRNA sequences to partial 16 S rRNA sequences in the database), we generated *in silico* partial sequences spanning the V1–4 and V4 hypervariable regions from the full-length PacBio 16 S rRNA sequences. Sequence assignment to the GG 16 S rRNA gene database resulted in close similarity of taxonomic profiles between full-length and partial sequences from the PacBio datasets (Supplementary Fig. 7). Assigning taxonomy of the GS FLX+, MiSeq V4, and PacBio datasets against the SILVA 16 S rRNA database resulted in overall higher proportion of assigned sequences when compared to those against the GG 16 S rRNA database (Supplementary Fig. 8a and b). In accordance with the assignment results based on the GG 16 S rRNA database (i.e., the lowest proportion of assigned sequences from the PacBio dataset, Fig. 2a), sequences assignment to the SILVA 16 S rRNA database at the genus level showed that the PacBio dataset had the lowest proportion of assigned sequences (mean 95.7, 95.6, and 93% for GS FLX+, MiSeq V4 and PacBio datasets, respectively). Assignment of the PacBio-generated full-length, and the *in silico* partial 16 S rRNA sequences to the SILVA 16 S rRNA database also showed no difference in the proportion of assigned sequences (Supplementary Fig. 8c and d), suggesting that differences in read length or region between query and subject sequences were not responsible for the reduced taxonomic resolution of the PacBio-generated human fecal bacterial sequences.

**Figure 4:**
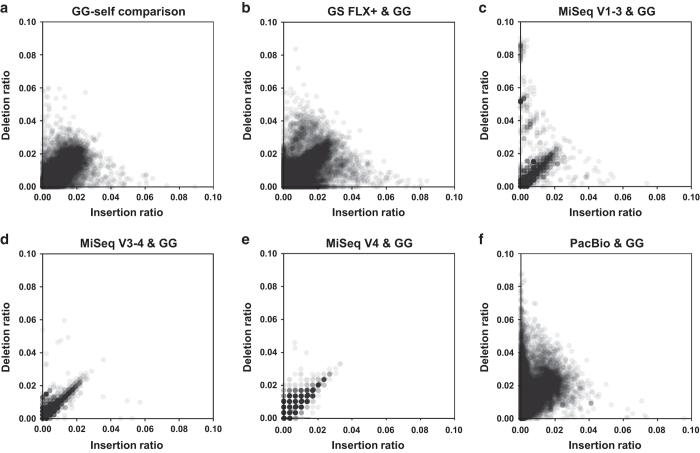
Profile of insertion and deletion errors within each platform-generated dataset. Plots of insertion and deletion abundance for (**a**) the GG 16 S rRNA gene database (self-comparison as a control) and the (**b**) GS FLX+, (**c**) Illumina MiSeq V1–3, (**d**) MiSeq V3–4, (**e**) MiSeq V4, and (**f**) PacBio datasets. The abundance of insertions (and deletions) was normalized against sequence length.

We next assessed the distribution of sequence similarities by assigning the 30,000 randomly extracted sequences from each platform against the GG 16 S rRNA gene database. We assumed no difference in the levels of similarity distribution among the sequences generated by each platform, if the extracted sequences contained no specific sequencing error(s). However, the results revealed that the PacBio and GS FLX+ datasets had significantly lower levels of mean similarity distribution than the MiSeq datasets (Fig. 3c). Interestingly, we observed significantly higher levels of mean similarity distribution in both the PacBio and GS FLX+ datasets after removing sequences containing insertion and deletion (indel) errors. The high level of indel errors in PacBio CCS sequences was confirmed by assessing error type(s) in publicly available PacBio-generated sequences (See Methods). Accordingly, we evaluated quantitative traits of insertion and deletion in the datasets generated by each platform by plotting insertion and deletion ratios. In contrast to the GS FLX+ and MiSeq datasets, which had linearly balanced distributions of insertion and deletion ratios, the PacBio datasets (both our data and data downloaded from public databases) had three times more plots weighted toward deletion (Fig. 4, and Supplementary Fig. 9). A summary of the error types (e.g., mismatch, insertion, and deletion) observed in each of the datasets is presented in terms of relative abundance in Supplementary Fig. 10.

### Sequencing performance of the MiSeq platform targeting the V1–3, V3–4, and V4 regions

Comparative analysis of GS FLX+-, MiSeq-, and PacBio-generated datasets demonstrated that the Illumina MiSeq exhibited high performance in terms of reflecting the shotgun data, and sequencing coverage. Accordingly, we focused our analysis on comparing three different amplification primer sets (V1–3, V3–4, and V4; *n*=165, for each dataset) generated by the Illumina MiSeq platform (Table 5). The major source of errors in Illumina data is related to substitution type miscalls, and the error frequency increases toward the ends of sequence reads^36,37^. Importantly, because the length of the target amplification regions increases in MiSeq paired-end sequencing, the error-prone regions of read 1 (R1) and read 2 (R2) are subjected to alignment to generate a single overlapped sequence. Thus, we speculated that the differences in read lengths obtained using different sets of primer pairs may affect the proportion of single merged sequences. The average lengths of the filtered paired-end reads (R1 and R2 reads) from the V1–3, V3–4, and V4 datasets were 243, 262, and 250 bp, respectively (Table 5). The quality-filtered paired-end reads were assembled using PEAR software, with default settings. Because the lengths of the merged sequences varied among datasets, we picked sequences in the ranges of 463–553 bp for the V1–3 dataset, 438–469 bp for the V3–4 dataset, and 286–298 bp for the V4 dataset, based on the length distributions of each of the target regions in the GG database (see Methods). As expected, the highest proportion of merged sequences was observed in the V4 dataset (98.2±1.7%), whereas the V1–3 dataset had the lowest efficiency of read assembly (14.2±3.4%) (Table 5). The quantitative features of the V1–3 dataset, followed by inefficient read assembly, resulted in lower coverage of human fecal microbiota (Supplementary Fig. 11a). Alpha diversity analyses comparing the MiSeq V1–3, V3–4 and V4 datasets also showed that all pairwise comparisons represented meaningful differences in diversity, richness, and evenness indices (Supplementary Fig. 11b). In particular, the richness indices (e.g., Chao1 and observed OTUs) in the V3–4 and V4 datasets were 2–3-fold higher than those in the V1–3 dataset.

### Sequence assignments of the MiSeq V1–3, V3–4 and V4 datasets

**Figure 5:**
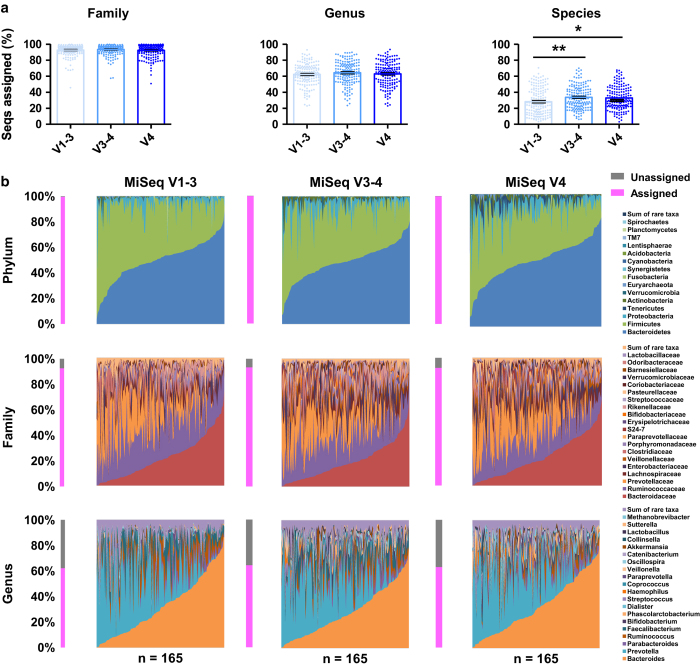
Taxonomy profiles of the Illumina MiSeq datasets. The proportion of assigned sequences (**a**) and relative abundance of the assigned sequences (**b**) from the MiSeq V1–3 (*n*=165, light blue), V3–4 (*n*=165, blue), and V4 (*n*=165, dark blue) datasets were determined by assigning sequences to the GG 16 S rRNA gene sequence database, and are represented at the family, genus, and species levels, respectively. Data were analyzed by ANOVA followed by Tukey’s *post-hoc* test (*P<0.05, **P<0.005, and ***P<0.001).

Next, we compared the proportions of assigned sequences by assigning the assembled sequences from each MiSeq dataset to the GG 16 S rRNA gene sequence database. We observed meaningful differences in proportions of the assigned sequences at the family or the genus levels (Fig. 5a). However, at the species level, the MiSeq V1–3 dataset had the lowest proportion of assigned sequences (mean 28.2, 33.7, and 32.8% for the V1–3, V3–4, and V4 datasets, respectively). Despite the poor efficiency of read assembly, the MiSeq V1–3 dataset had the longest read length of the assembled sequences among the three MiSeq-generated datasets. These data, along with the low proportion of assigned sequences in the PacBio dataset, in which longer reads are abundant (Fig. 2a), suggest that sequencing error is the result of low taxonomic resolution in the long read abundant datasets.

### Phylogenetic analyses of the MiSeq V1–3, V3–4, and V4 datasets

**Figure 6:**
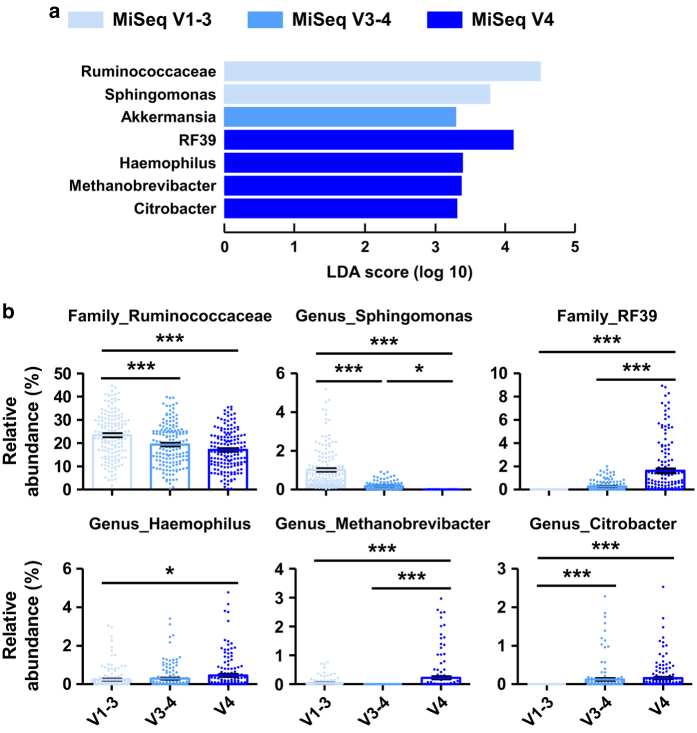
Abundance patterns of bacterial taxa in Illumina MiSeq datasets. (**a**) The abundance patterns of bacterial taxa in the MiSeq V1–3 (*n*=165, light blue), V3–4 (*n*=165, blue), and V4 (*n*=165, dark blue) datasets were analyzed according to linear discriminant analysis effect size (LEfSe). (**b**) The relative abundance of the families *Ruminococcaceae* and *RF39*, and the genera *Sphingomonas*, *Haemophilus*, *Methanobrevibacter*, and *Citrobacter* in OTU tables for each dataset are represented as bar graphs. Data were analyzed by ANOVA, followed by Tukey’s *post-hoc* test (*P<0.05, **P<0.005, and ***P<0.001).

Despite the differences in alpha diversity indices and efficiency of read assembly in the MiSeq datasets described above, the relative abundance of the 165 human fecal microbial communities showed remarkably similar patterns at the phylum, family, and genus levels (Fig. 5b). We next specified the abundance patterns of bacterial taxa in each MiSeq dataset using LEfSe analysis. The effect size estimations of LEfSe, combined with the relative abundance of microbial taxa in OTU tables, indicated that the family *Ruminococcaceae* and genus *Sphingomonas* were discriminant taxa for the MiSeq V1-3 dataset; genus *Akkermansia* was the discriminant taxon for the MiSeq V3–4 dataset; and family *RF39* and genera *Haemophilus*, *Methanobrevibacter*, and *Citrobacter* were the discriminant taxa for the MiSeq V4 dataset (Fig. 6). These results were based on the sub-sampled sequences by the samples possessing the minimal number of sequences in each dataset (i.e., 3,279, 21,313, and 42,841 reads for the V1–3, V3–4, and V4 datasets, respectively). To minimize sample size-induced bias between the datasets, we rarefied all samples by sub-sampling 3,000 sequences. Alpha diversity analyses of the 3,000 sub-sampled datasets revealed comparable or even significantly lower values for the diversity, richness, and evenness indices for the V3–4 and V4 datasets than for the V1–3 dataset (Supplementary Fig. 12a), suggesting overestimation of richness values in the non-rarefied V3–4 and V4 datasets (Supplementary Fig. 11). Despite meaningful changes in alpha diversity indices, the taxonomic profiles of fecal microbial communities yielded similar patterns at the phylum and genus levels (Supplementary Fig. 12b).

## Usage Notes

We expected that full-length sequences of 16 S rRNA genes, rather than partial gene sequences, would be advantageous for inferring phylogenetic affiliations because long-read sequencing is capable of covering a large portion of the target gene, thereby potentially increasing the resolution with which one can discriminate many phylogenetically closely related taxa^38–40^. In this regard, the ability of PacBio CCS to produce long sequence reads (2–15 kb) has focused a great deal of attention on this platform as a replacement for Sanger sequencing, which generates nearly full-length 16 S rRNA gene sequences with expense of low throughput scale^20^. Indeed, several comparative studies have indicated that the suitability of the PacBio CCS for 16 S rRNA gene sequencing has incrementally increased over the past several years: the error rate of PacBio sequencing is now comparable with that of the shorter reads produced by the 454 and MiSeq platforms^23^, OTU inference has improved^40^, and phylogenetic resolution of microbial communities has become more accurate^20^. However, a study focused on homology detection in human medical amplicon data raised concerns that the most frequent errors in PacBio-generated sequences are indels^41^, which can affect microbial abundance profiling^42^. Our results are consistent with this, and reveal the error types observed in practice when using the PacBio CCS for a scaled-up study of the complex microbial community in human fecal samples. The connection between indel errors and the results of taxonomic assignment (i.e., the predominance of the phylum *Proteobacteria*) in the PacBio dataset remains unclear. Collectively, the data suggest that continuous improvement of the PacBio system in terms of accuracy and sequencing quantity, together with the results of sequencing error profiling studies, make the long read sequencer a promising tool for full-length and high-resolution 16 S rRNA gene profiling of human gut microbiota.

It is also worth mentioning here that the taxonomic resolution in amplicon sequencing might be affected by several additional factors, including the primer specificity^19,24^, choice of hypervariable region^43,44^, reference database^34^, and environmental source of sample^39^. In our analysis, we generated GS FLX+ (V1–4), Illumina MiSeq (V1–3), and PacBio CCS (V1–9) datasets from fecal samples. In this case, Illumina MiSeq dataset contained overlapping region, whereas GS FLX+ and PacBio CCS datasets included both overlapping and non-overlapping regions. Therefore, we do not rule out the possibility that differences in primer specificity, read length, hypervariable region, and amplification fidelity may lead to differences in the amplification efficiency, and further taxonomic abundance estimation.

Owing to its technically amenable read length for the Illumina system, the V4 region (as shown in the comparative analysis of the GS FLX+ and PacBio systems (Fig. 2)) has been used widely for sequencing amplicons derived from environmental 16 S rRNA genes^15,45^. However, the increase in sequence read length by the MiSeq platform has enabled users to choose several additional primer pair combinations to cover longer regions of 16 S rRNA hypervariable regions. This issue has not been of great interest, however, because the 454 series of platforms have been used for amplicon sequencing for a long time, and reagents for extending read length (2×300 bp) became available quite recently. Our comparative analysis of the MiSeq paired-end sequencing data revealed that targeting the V1–3 region of human gut microbiota is advantageous for increasing the read length (mean, 516±14 bp), whereas the low efficiency in terms of read assembly results in low sequencing coverage of a sample. At the species level, the MiSeq V1-3 dataset had the lowest proportion of assigned sequences. It may be possible that the V1-3 overlapped/middle region is the lowest quality due to enrichment of errors at the 3′ termini of the sequences from both of the primers. This high error rate in the middle of V1-3 sequence may cause the lowest of assigned sequences to the GG 16 S rRNA database. Targeting the V3–4 region represents an attractive alternative to fragment sequencing of human fecal 16 S rRNA genes because the MiSeq V3–4 dataset shows the strongest closest relationship with the shotgun data, generates much longer merged sequences than those of the V4 dataset, and shows higher efficiency in terms of read assembly (comparable with that of the V4 dataset). To keenly discriminate the closely related taxa in human gut microbiota, the target region and subsequent choice of primer pair must vary with increased sequencing accuracy and read length. In this context, targeting the V3–4 region of the MiSeq paired-end application (with the reagent kit v3) currently represents the ideal balance point between quantitative and qualitative characteristics.

## Additional information

**How to cite this article:** Whon, T.W. *et al.* The effects of sequencing platforms on phylogenetic resolution in 16S rRNA gene profiling of human feces. *Sci. Data* 5:180068 doi: 10.1038/sdata.2018.68 (2018).

**Publisher’s note:** Springer Nature remains neutral with regard to jurisdictional claims in published maps and institutional affiliations.

## Supplementary Material



Supplementary Information

## Figures and Tables

**Table 1:** Summary of sequencing data generated by each sequencing platform.

**Platform**	**Target region**	**Source**	**Sample size**	**Project**	**Study**	**Base**
GS FLX+	V1-4	Human feces	170	PRJEB17507	ERP019369	8.2 G
MiSeq	V1-3	Human feces	170	PRJEB17608	ERP019474	6.8 G
MiSeq	V3-4	Human feces	170	PRJEB17610	ERP019476	7.3 G
MiSeq	V4	Human feces	170	PRJEB17613	ERP019478	7.5 G
PacBio	V1-9	Human feces	29	PRJEB17612	ERP019477	5.1 G
HiSeq	Whole-metagenome	Human feces	27	PRJEB17896	ERP019800	298 G

**Table 2:** Spearman’s rank correlation coefficients and corresponding *P*-values.

	**MiSeq V4**	**MiSeq V1-3**	**MiSeq V3-4**	**GS FLX+ V1-4**	**PacBio**
**Shotgun 16 s**	0.97 (1.935E-16)	0.75 (5.439E-06)	0.98 (4.126E-18)	0.89 (5.203E-10)	0.79 (7.658E-07)
**MiSeq V4**		0.74 (1.255E-05)	0.98 (5.737E-19)	0.83 (7.267E-08)	0.77 (2.778E-06)
**MiSeq V1-3**			0.79 (1.238E-06)	0.78 (1.507E-06)	0.60 (1.038E-03)
**MiSeq V3-4**				0.86 (8.045E-09)	0.75 (6.036E-06)
**GS FLX+**					0.80 (4.688E-07)

**Table 3:** Summary of the GS FLX+, MiSeq V4, and PacBio datasets.

	**GS FLX+**	**MiSeq V4**	**PacBio**
Read quantity	4 regions	A flow cell	4 cells
No. of samples	169	169	29
Raw sequence reads (mean reads per sample)	1,322,495 (7,825)	15,011,155 (88,823)	118,289 (4,079)
Quality filtered	1,127,106	14,730,475	97,816
Chimera removed	793,845	13,988,284	83,913
Singleton removed	742,576	13,260,737	76,814
No. of seqs per samples (mean)	2,137 - 42,563 (4,394)	42,838 - 167,506 (78,466)	1,395 - 5,610 (2,649)
Sub-sampled	2,137	42,838	1,395
Mean read length (bp)	646±141	291	1,481±27

**Table 4:** Summary of the subject-standardized (*n*=29) GS FLX+, MiSeq V4, and PacBio datasets.

	**GS FLX+**	**MiSeq V4**	**PacBio**
No. of samples	29	29	29
Raw sequence reads (mean reads per sample)	235,007 (8,104)	2,609,369 (89,978)	118,289 (4,079)
Quality filtered	201,554	2,571,350	97,816
Chimera removed	138,553	2,443,968	83,913
Singleton removed	128,593	2,311,742	76,814
No. of seqs per samples (mean)	2,085 - 5,870 (4,434)	62,637 - 167,192 (79,715)	1,395 - 5,610 (2,649)
Sub-sampled	2,085	62,637	1,395
Mean read length (bp)	657±146	291	1,481±27

**Table 5:** Summary of the MiSeq V1–3, V3–4, and V4 datasets.

	**MiSeq V1-3**	**MiSeq V3-4**	**MiSeq V4**
Read quantity	A flow cell	A flow cell	A flow cell
No. of samples	165	165	165
Raw sequence reads (R1+R2)	22,347,080	23,654,914	29,406,440
Filtered reads (R1+R2)	21,083,242	23,427,732	29,147,046
Mean lengths of the filtered paired-end reads (bp)	243±27	262±18	250±0.7
No. of merged sequences	1,491,506	10,261,500	14,434,301
Assembly efficiency (%)	14.2±3.4	87.6±2.3	98.2±1.7
Chimera removed	1,302,039	9,166,941	13,705,970
Singleton removed	1,123,787	8,494,242	12,994,272
No. of seqs per samples (mean)	3,279 - 14,995 (6,811)	21,313 - 125,775 (51,480)	42,841 - 167,478 (78,543)
Sub-sampled	3,279	21,313	42,841
Mean read length (bp)	516±14	454±10	291
