# Supplementary material for: The effects of sequencing platforms on phylogenetic resolution in 16 S rRNA gene profiling of human feces
Source: Sci Data. 2018 Apr 24;5:180068. doi: 10.1038/sdata.2018.68 (PMC5914283; doi:10.1038/sdata.2018.68)
Supplement: Supplementary Information [file sdata201868-s2.doc]

**Supplementary information for**

**The effects of sequencing platforms on phylogenetic resolution in 16S rRNA gene profiling of human feces**

Tae Woong Whon^1†^, Won-Hyong Chung^2†^, Mi Young Lim^2^, Eun-Ji Song^2,3^, Pil Soo Kim^1^, Dong-Wook Hyun^1^, Na-Ri Shin^1^, Jin-Woo Bae^1^*, and Young-Do Nam^2,3^*

^1^Department of Life and Nanopharmaceutical Sciences and Department of Biology, Kyung Hee University, Seoul 130-701, Republic of Korea

^2^Research Group of Gut Microbiome, Division of Nutrition and Metabolism Research, Korea Food Research Institute, Sungnam 463-746, Republic of Korea

^3^Korea University of Science and Technology, Daejeon 305-350, Republic of Korea

^†^Equal contributors

*Correspondence: **baejw@khu.ac.kr**; [youngdo98@kfri.re.kr](mailto:youngdo98@kfri.re.kr)


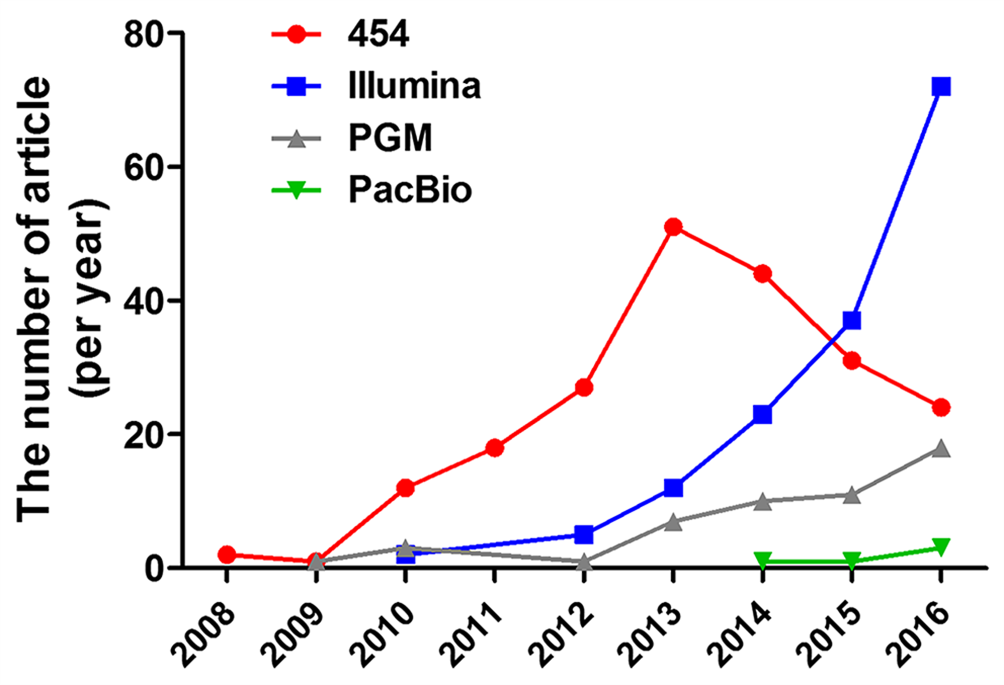


**Supplementary Figure 1. Overview of human gut microbial studies conducted using next-generation sequencing.** The annual number of publications, determined by Thomson Innovation Literature Export searches (http://thomsonreuters.com), using the keywords ‘ALL=((human* or adult* or child* or bab* or elder* or patient* or individual* or rac* or nation*) AND (gut or intestinal* or intestine* or fecal* or fece* or stool*) AND (16S or rRNA or rDNA or ribosomal*) AND (microbiome or microbiota or flora* or bacteria*) AND (pgm or ion or torrent or proton)) AND (TF>=(2006) AND TF<=(2016)) AND DT=("Article")’ is shown on the *y*-axis. Metagenomics studies conducted by 454, Illumina, PGM, and PacBio systems are shown in red, blue, grey, and green, respectively.

**
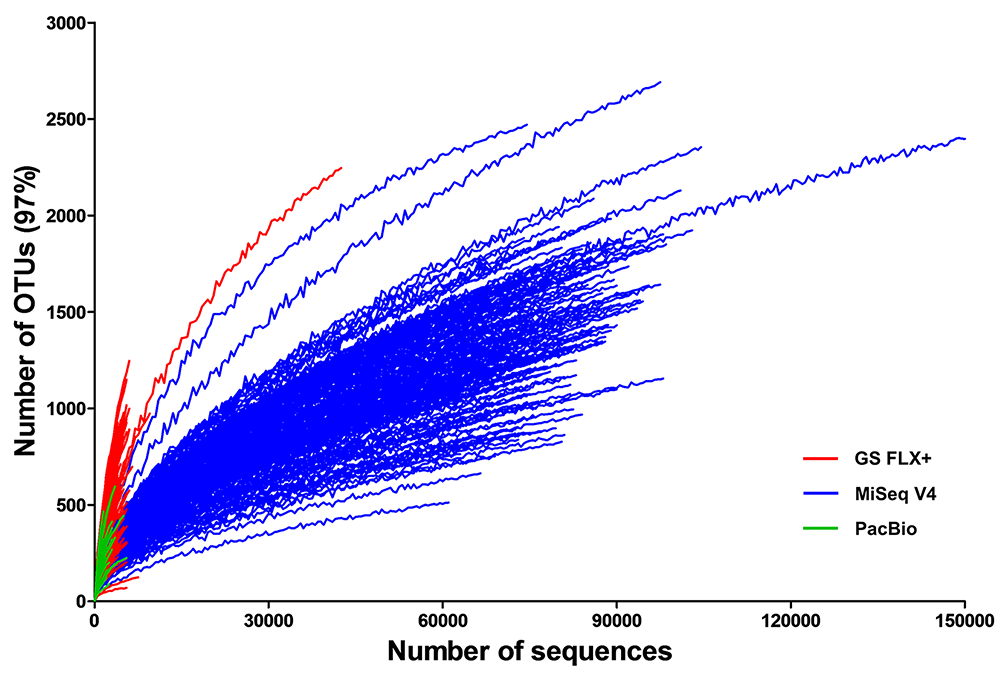
**

**GS FLX+**

**MiSeq V4**

**PacBio**

**Supplementary Figure 2. Rarefaction analysis of human fecal sequences generated by the GS FLX+, Illumina MiSeq and PacBio systems.** Fecal samples collected from human subjects were sequenced by GS FLX+ (n=169, V1–4, red), Illumina MiSeq (n=169, V4, dark blue) and PacBio CCS (n=29, V1–9, green). Rarefaction curves were calculated at a 3% sequence dissimilarity level.


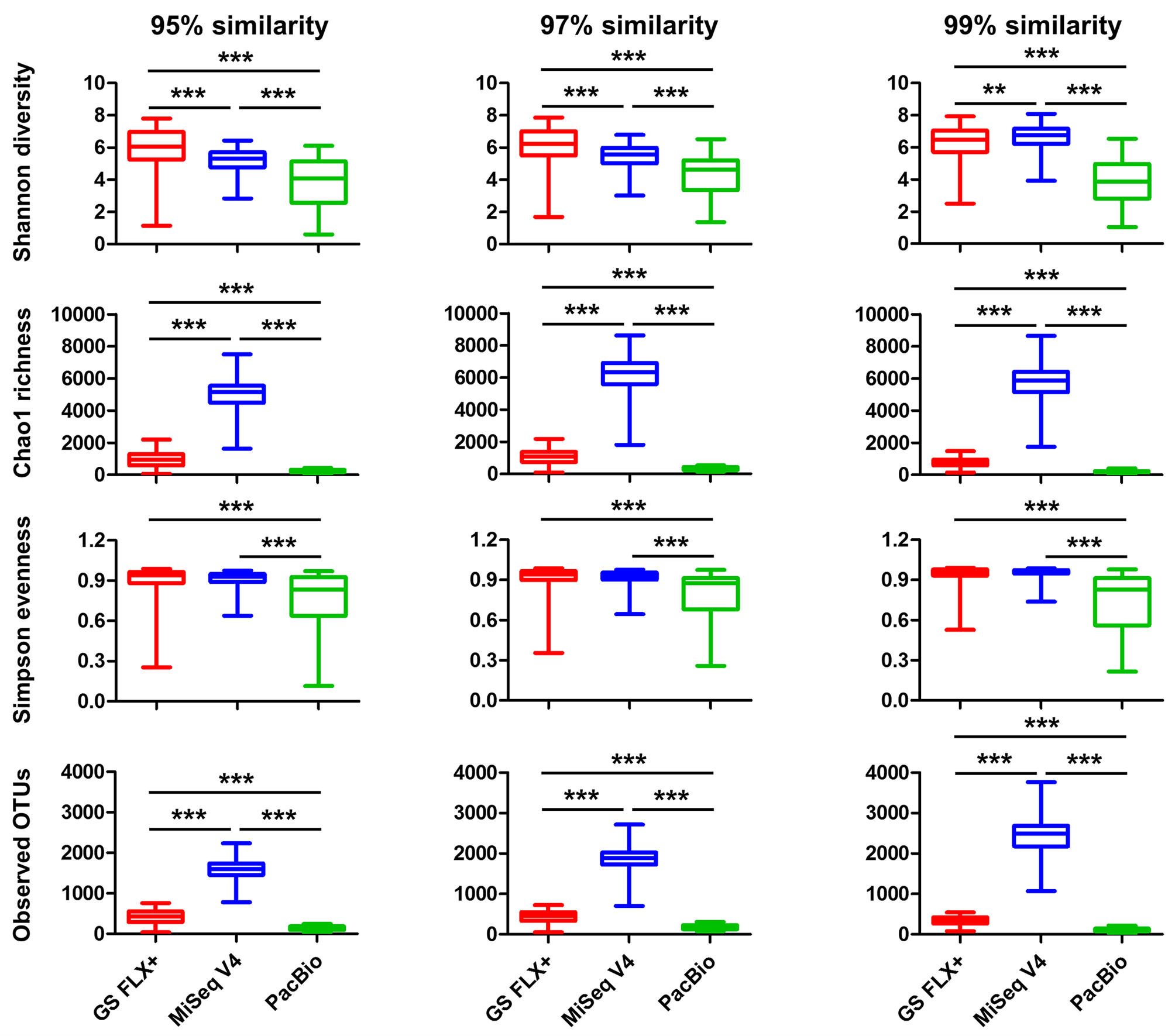


**Supplementary Figure 3. Alpha diversity analyses of human fecal sequences generated from GS FLX+, Illumina MiSeq, and PacBio systems.** Fecal samples collected from human subjects were sequenced by GS FLX+ (n=169, V1–4, red), Illumina MiSeq (n=169, V4, dark blue) and PacBio CCS (n=29, V1–9, green). Alpha diversity indices (Shannon diversity, Chao1 richness, Simpson evenness, and observed OTUs) were calculated at 1, 3, and 5% sequence dissimilarity levels, respectively. Data were analyzed by ANOVA, followed by Tukey’s post hoc test (*p < 0.05, **p < 0.005, and ***p < 0.001).


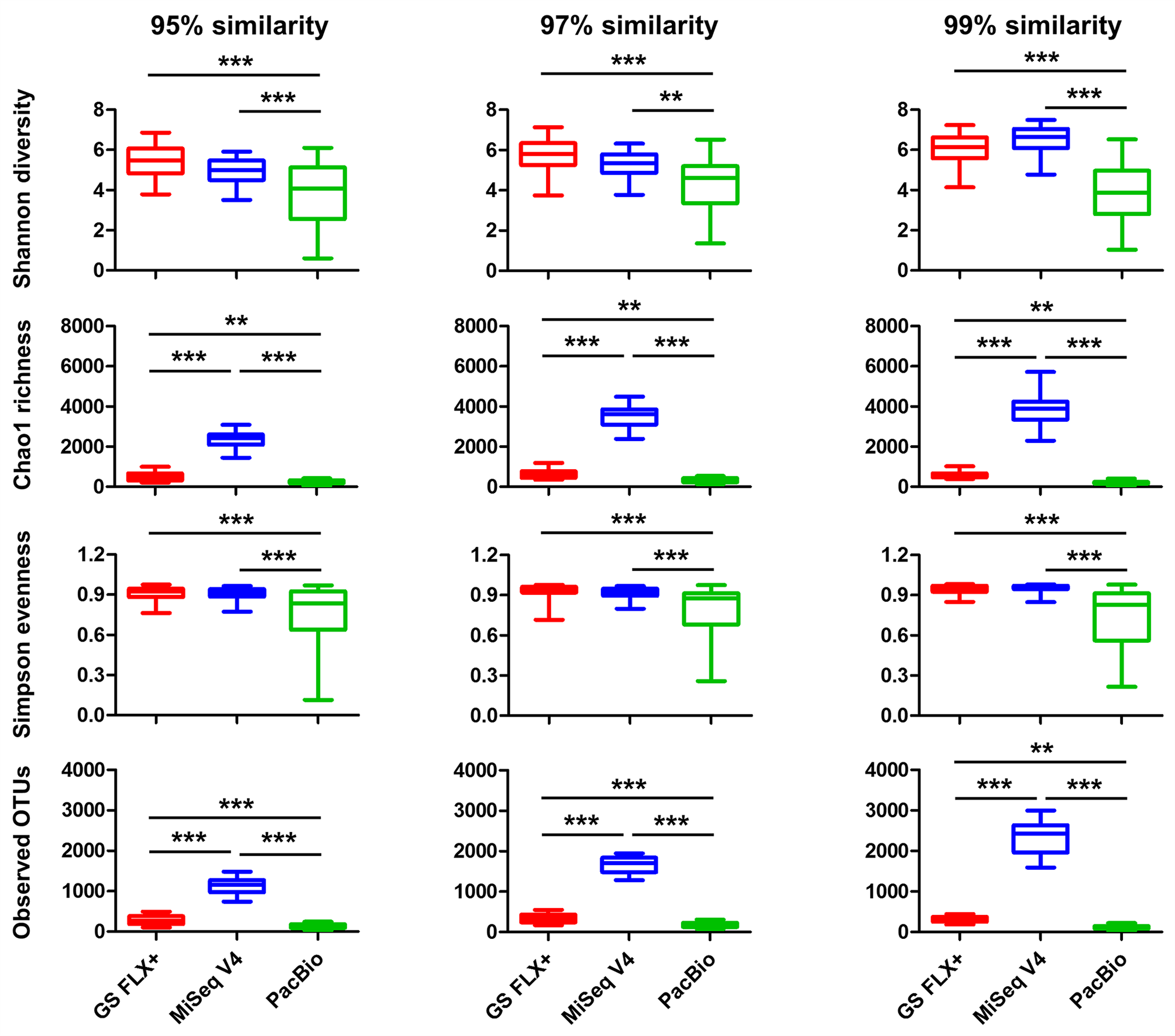


**Supplementary Figure 4. Alpha diversity analyses of subject-standardized GS FLX+, MiSeq, and PacBio datasets.** Fecal samples collected from 29 human subjects were sequenced by GS FLX+ (V1–4, red), Illumina MiSeq (V4, dark blue) and PacBio CCS (V1–9, green). Alpha diversity indices (Shannon diversity, Chao1 richness, Simpson evenness, and observed OTUs) were calculated at 1, 3, and 5% sequence dissimilarity levels, respectively. Data were analyzed by ANOVA, followed by Tukey’s post hoc test (*p < 0.05, **p < 0.005, and ***p < 0.001).


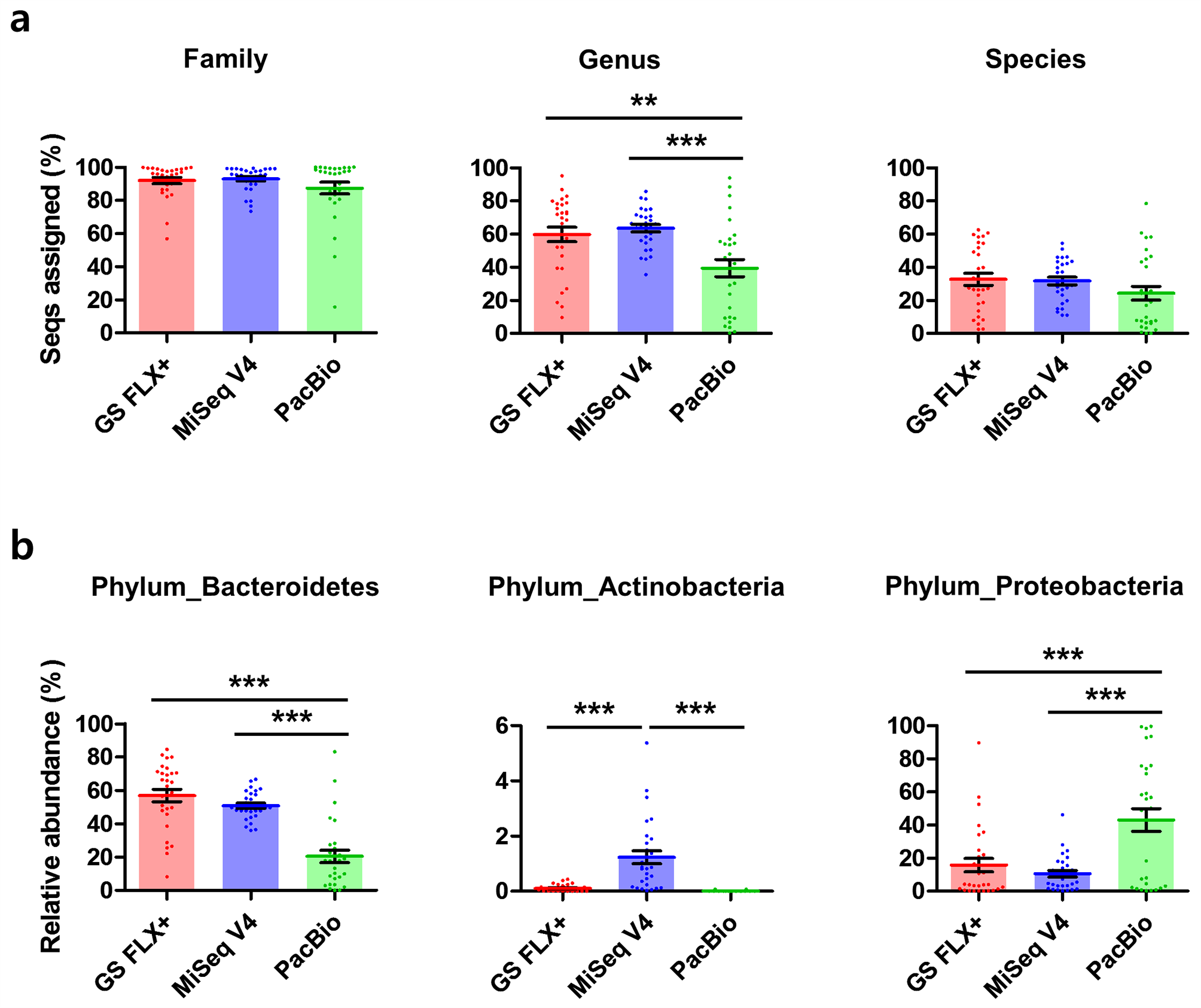


**Supplementary Figure 5. Taxonomy profiles in subject-standardized GS FLX+, MiSeq, and PacBio datasets.** Fecal samples collected from 29 human subjects were sequenced by GS FLX+ (V1–4, red), Illumina MiSeq (V4, dark blue) and PacBio CCS (V1–9, green). (a) The proportion of assigned sequences from each dataset was determined by assigning the sequences to the Greengenes 16S rRNA gene sequence database, and is represented at the family, genus, and species levels. (b) The relative abundances of phyla *Bacteroidetes*, *Actinobacteria* and *Proteobacteria* in OTU tables of each dataset are represented as bar graphs. Data were analyzed by ANOVA, followed by Tukey’s post hoc test (*p < 0.05, **p < 0.005, and ***p < 0.001).


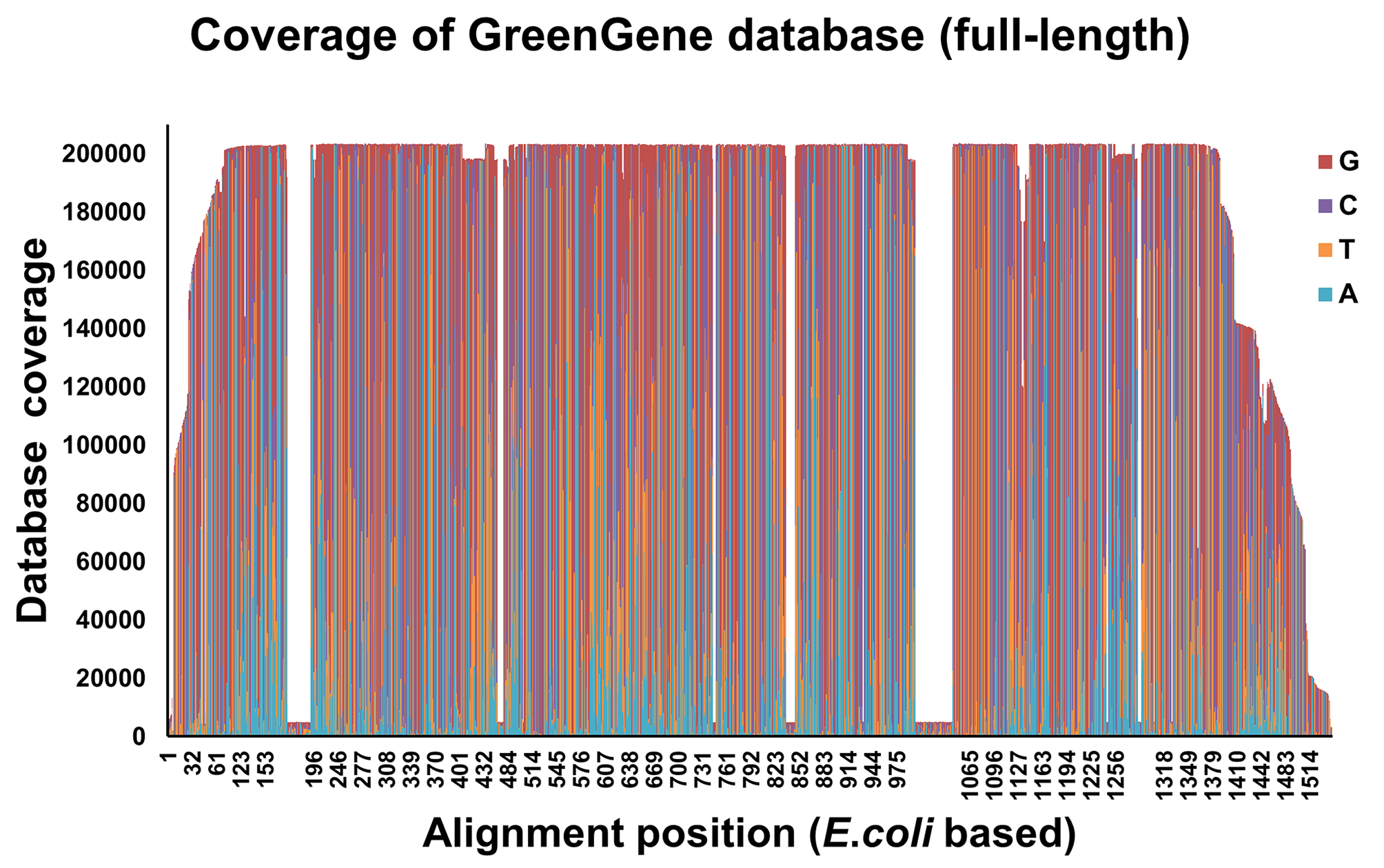


**Supplementary Figure 6. Coverage of 16S rRNA genes in the Greengenes database.** In total, 203,452 sequences obtained from the Greengenes (GG) database (gg_13_8_99) were aligned with 16S rRNA genes of *Escherichia coli*. The x and y axes represent alignment position and database coverage, respectively.


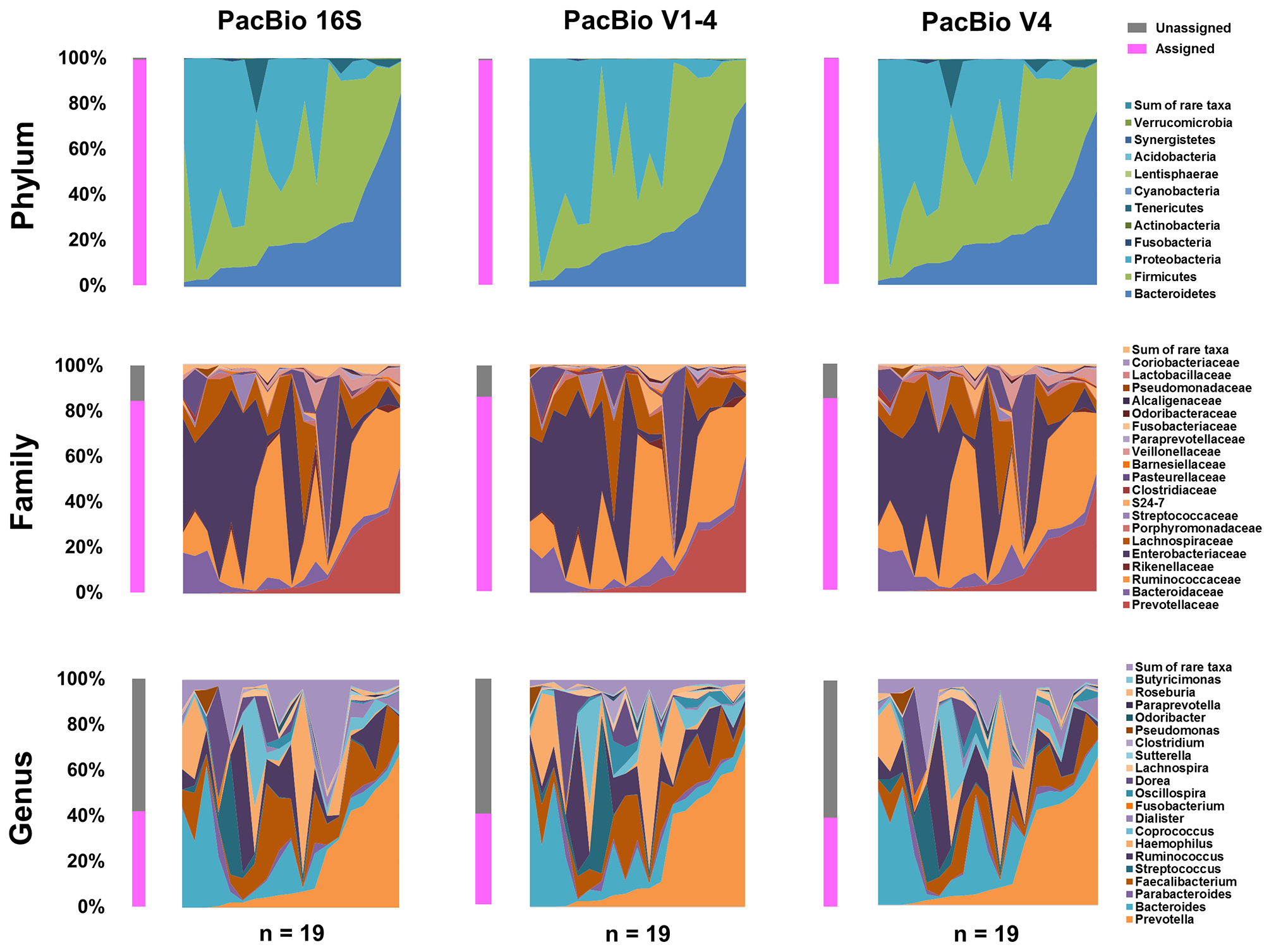


**Supplementary Figure 7. Taxonomy profiles in PacBio datasets generated *in silico*.** Partial 16S rRNA gene sequences spanning V1–4 and V4 regions were generated *in silico* from full length of the PacBio (n=29) datasets. The relative abundances of the assigned sequences from the PacBio full-length, V1–4, and V4 datasets were determined by assigning the sequences to the Greengenes 16S rRNA gene sequence database, and are represented at the family, genus, and species levels.


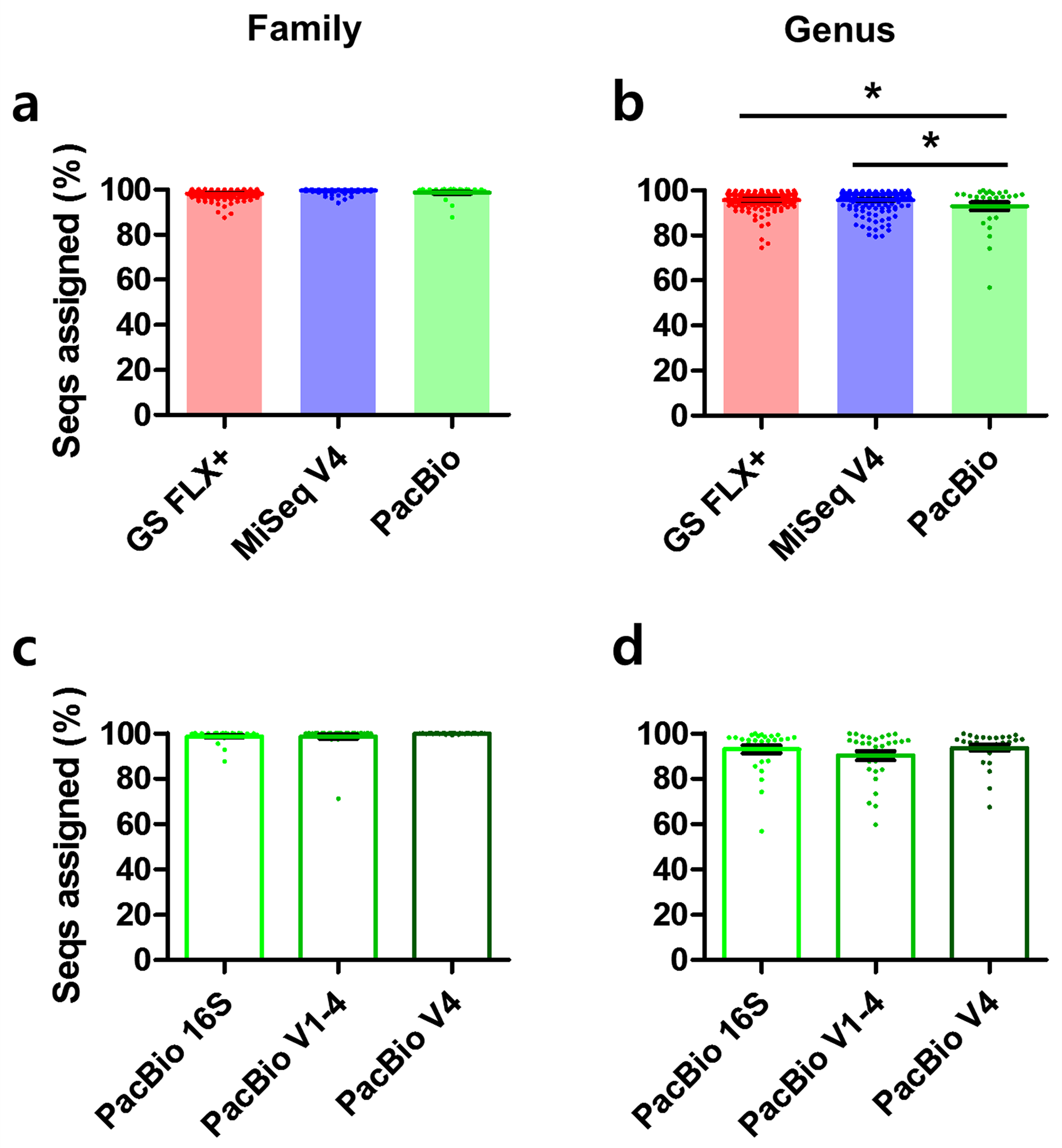


**Supplementary Figure 8. Taxonomy profiles against the SILVA 16S rRNA database.** (a and b) The proportion of assigned sequences from the GS FLX+ (n=169), MiSeq V4 (n=169), and PacBio (n=29) datasets was determined by assigning the sequences to the SILVA 16S rRNA database, and are represented at the family (a), and genus (b) levels. (c and d) The proportion of assigned sequences from the PacBio full-length, V1–4, and V4 datasets (n=29, for each dataset) was determined by assigning the sequences to the SILVA 16S rRNA database, and are represented at the family (c), and genus (d) levels.


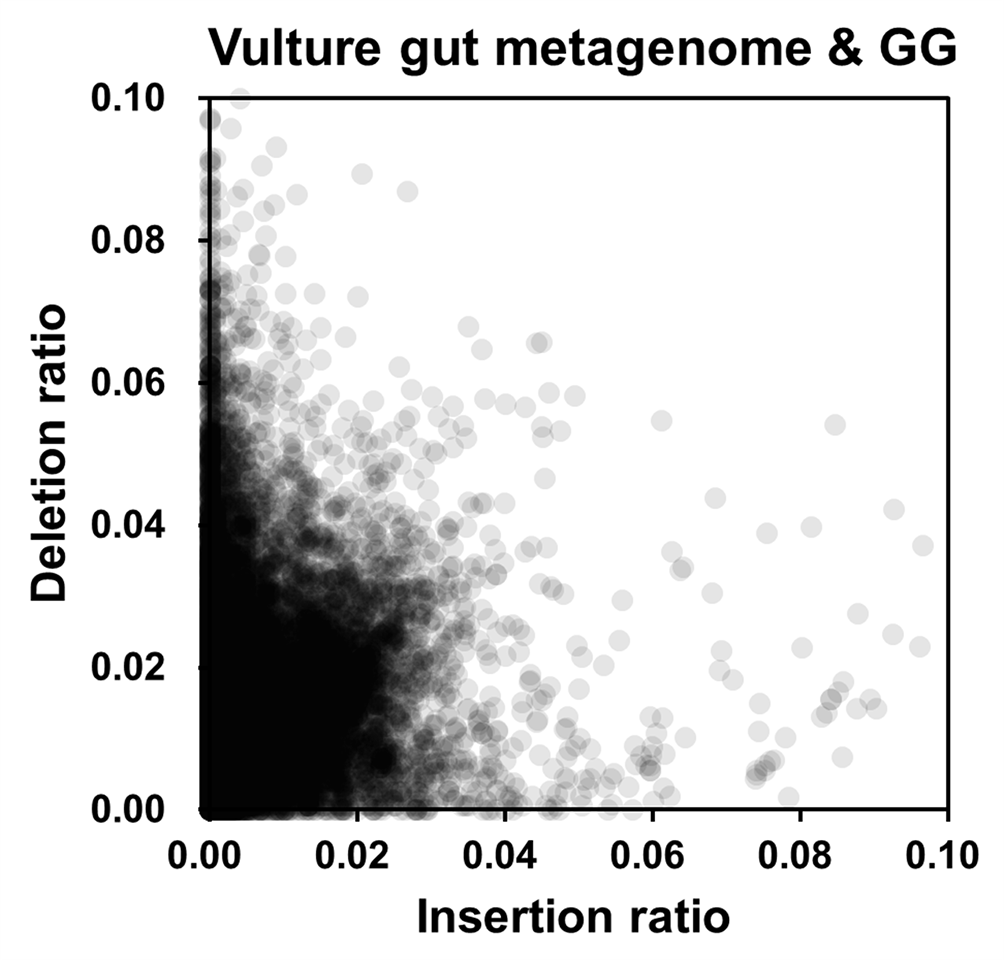


**Supplementary Figure 9. Profile of insertion and deletion errors in publicly available PacBio sequences.** Sequences (n=30,000) were randomly sub-sampled from publicly available PacBio sequences (vulture gut metagenome, SRR4033078). The sub-sampled sequences were aligned with the GG 16S rRNA gene database. Insertion and deletion ratios in each sub-sampled sequence are represented as a scatter plot.


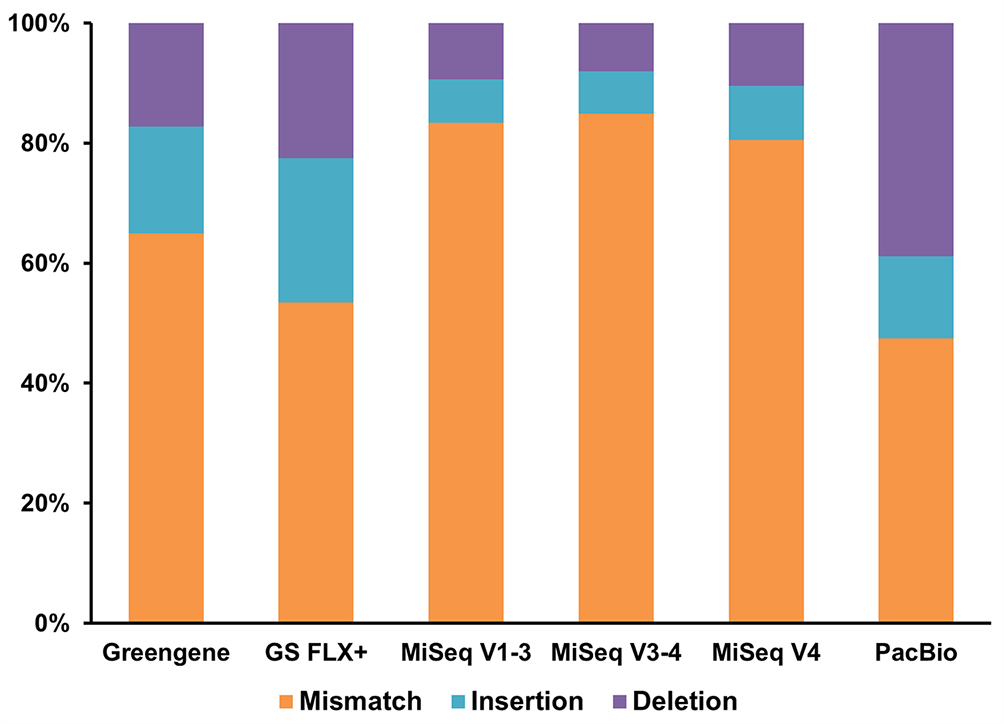


**Supplementary Figure 10. Summary of error types in datasets generated by each platform.** Sequences (n=30,000) were randomly sub-sampled from the GG 16S rRNA gene database (self-comparison as a control), GS FLX+, Illumina MiSeq V1–3, MiSeq V3–4, MiSeq V4, and PacBio datasets. Types of variations (e.g., mismatch, insertion, and deletion) were calculated based on inter-comparisons of sequence dissimilarities between sub-sampled sequences and the GG 16S rRNA gene database, and are represented as relative abundance.


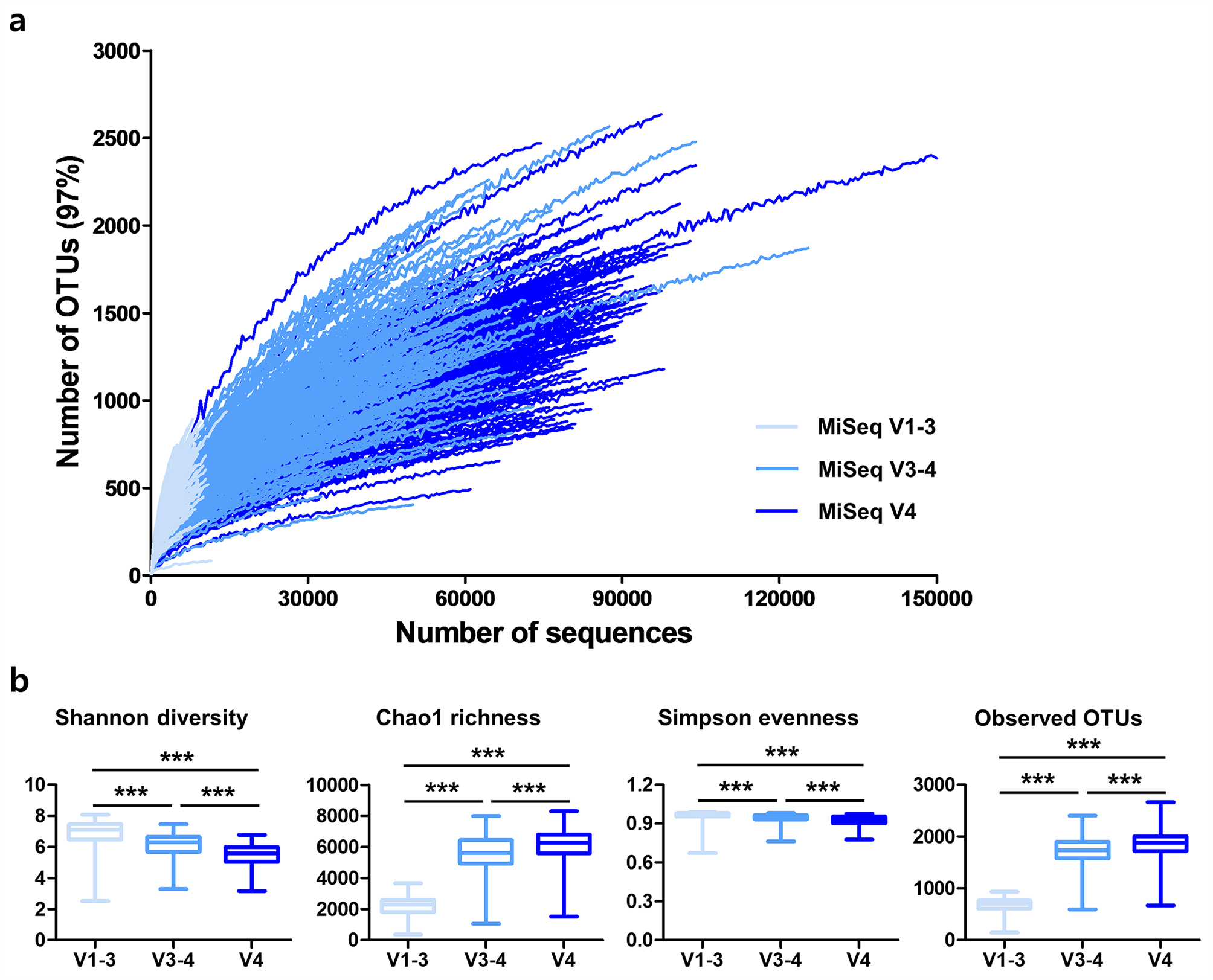


**Supplementary Figure 11. Rarefaction and alpha diversity analysis of human fecal sequences generated from Illumina MiSeq V1–3, V3–4, and V4 datasets.** Fecal DNA collected from 165 human subjects was amplified using three different amplification primer sets (V1–3, light blue; V3–4, blue; and V4, dark blue), and sequenced on the Illumina MiSeq platform. (a) Rarefaction curves and (b) alpha diversity indices (Shannon diversity, Chao1 richness, Simpson evenness, and observed OTUs) were calculated at a 3% sequence dissimilarity level. Data were analyzed by ANOVA, followed by Tukey’s post hoc test (*p < 0.05, **p < 0.005, and ***p < 0.001).


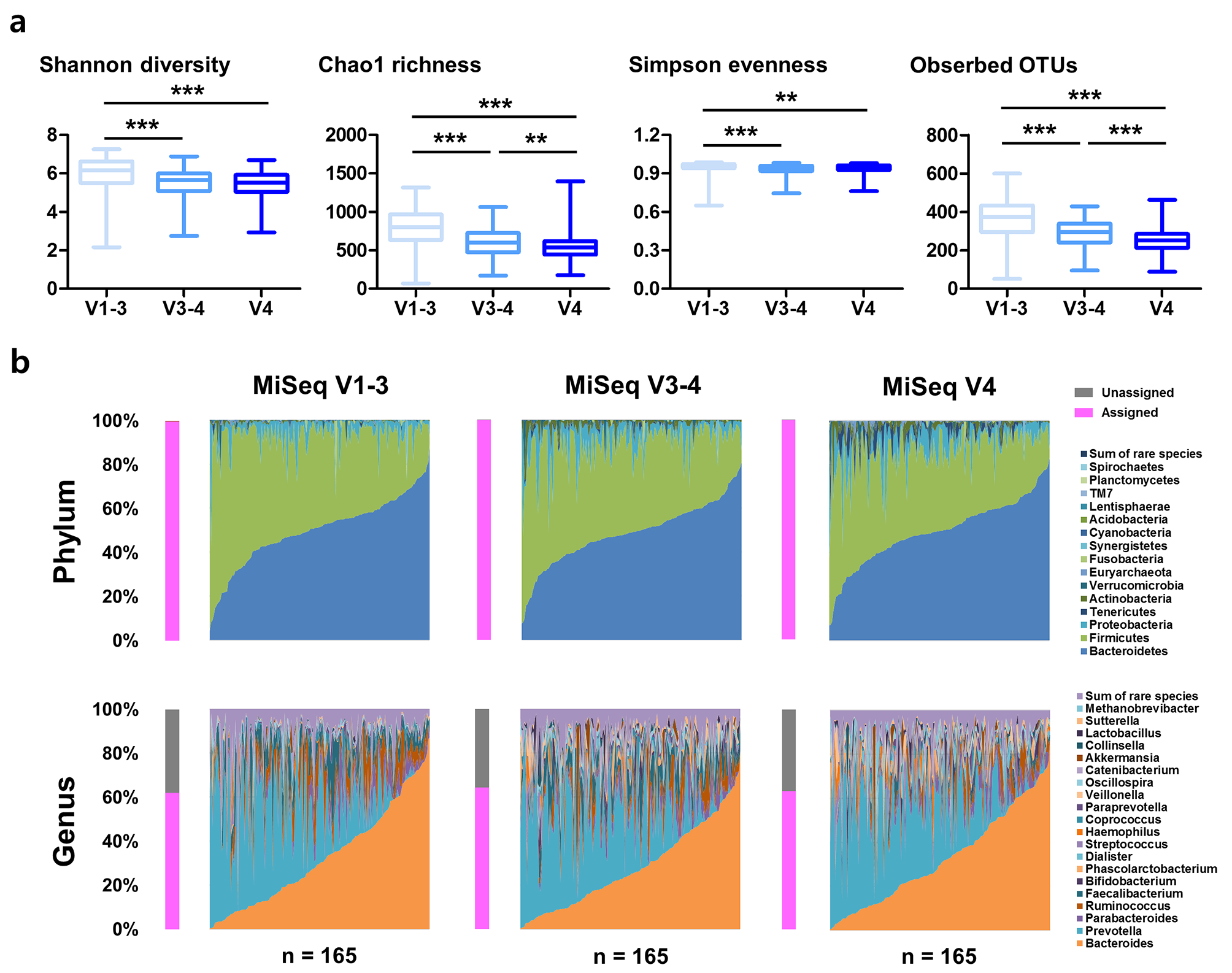


**Supplementary Figure 12. Alpha diversity analyses and taxonomy profiles in 3,000 sub-sampled Illumina datasets.** Illumina datasets generated using three different amplification primer sets (V1–3, V3–4, and V4) were randomly sub-sampled in sets of 3,000 sequences. (a) Alpha diversity indices (Shannon diversity, Chao1 richness, Simpson evenness, and observed OTUs) were calculated at a 3% sequence dissimilarity level. (b) Relative abundances of assigned sequences from the sub-sampled Illumina datasets were determined by assigning the sequences to the Greengenes 16S rRNA gene sequence database, and are represented at the family, genus, and species levels. Data were analyzed by ANOVA followed by Tukey’s post hoc test (*p < 0.05, **p < 0.005, and ***p < 0.001).
